# Current status and influencing factors of spiritual needs of patients with advanced cancer: a cross-sectional study

**DOI:** 10.1186/s12912-023-01306-9

**Published:** 2023-04-19

**Authors:** Xin Shi, Fengxia Wang, Lixin Xue, Zhaohong Gan, Yan Wang, Qian Wang, Xiaorong Luan

**Affiliations:** 1 Department of Oncology, Fourth People’s Hospital of Jinan City, Shandong, China; 2 School of Nursing and Rehabilitation, Shandong University, Jinan City, Shandong Province China; 3 Department of Oncology, Zaozhuang Municipal Hospital, Shandong, China; 4 Department of Oncology, Shandong Cancer Hospital and Institute, Shandong, China; 5 Department of Infection Control, Qilu Hospital of Shandong University, Jinan City, Shandong Province China

**Keywords:** Cancer, Spirituality, Spiritual needs, Cancer-related fatigue, Family care index, Social support, Root cause analysis

## Abstract

**Background:**

Spiritual needs have been associated with better physical health outcomes and provide a context for patients to gain hope and significance in coping with disease. This study aimed to understand the status of spiritual needs of patients with advanced cancer and conducted a quantitative study on the relationship between patient-reported physical, psychological, and social influencing factors and spiritual needs based on a biopsychosocial-spiritual model.

**Methods:**

In this study, 200 oncology inpatients from Shandong Province were recruited using a convenience sampling method to conduct a cross-sectional survey using general data from December 2020 to June 2022. Correlation analysis was used to analyze the correlation between spiritual needs and cancer-related fatigue, anxiety and depression, the family care index, and social support. Multiple regression analysis was used to analyze the relationship between spiritual needs and the influencing factors.

**Results:**

The spiritual needs score of the patients with advanced cancer was high. Multiple regression analysis revealed that cancer-related fatigue, social support, and religious beliefs influenced the spiritual needs of patients with advanced cancer. Compared with married patients, widowed or divorced patients scored 8.531 points higher on spiritual needs. Cancer-related fatigue, social support, religious beliefs, and marital status (divorced or widowed) explained 21.4% of the total variation in the spiritual needs of patients with advanced cancer.

**Conclusion:**

The spiritual needs of patients with advanced cancer were significantly correlated with cancer-related fatigue, depression, social support, and other factors. Religious beliefs, marital status, cancer-related fatigue, and social support were the main factors influencing the spiritual needs of patients with advanced cancer. This is a quantitative study, and medical staff can provide targeted spiritual care for patients with cancer based on the above influencing factors.

**Supplementary Information:**

The online version contains supplementary material available at 10.1186/s12912-023-01306-9.

## Background

Cancer has become a major public health problem that seriously threatens the lives and health of all human beings worldwide [1]. According to the latest global data released by the International Agency for Research on Cancer (IARC) of the World Health Organization, there have been nearly 20 million new cancer cases worldwide and nearly 10 million cancer-related deaths in 2020. In China, new cancer cases account for 23.7% of new global cases, and deaths account for 30.2% of global cancer-related deaths, both of which rank first worldwide [2]. In the future, the double burden of infection- and lifestyle-related cancers will increase the overall morbidity and mortality of cancers in China [3]. With the continuous improvement in medical techniques and effective treatment, the 5-year survival rate of patients with cancer has greatly improved. Cancer is currently regarded as a chronic disease. Cancer symptoms, as well as the long-term and repeated treatment process, not only damage the basic physiological functions of the patients to varying degrees, but also cause them to experience huge mental and psychological trauma and pain [4]. Approximately one in five patients in palliative care were identified as “spiritually distressed” [5], defined as disappointment, powerlessness, loneliness, and depression. Simultaneously, as cancer progresses, physical functioning deteriorates and symptoms, including fatigue, dyspnea, nausea, and pain, tend to increase [6]. These physical and emotional symptoms exacerbate existential concerns related to meaning and purpose, independence, abandonment, and dignity [7]. Spirituality comes into focus when an individual faces emotional stress, physical illness, or death [8]. Spirituality may provide a context in which people can make sense of their lives and feel whole, hopeful, and peaceful even in the midst of life’s most serious challenges [9]. Evidence indicates that spiritual distress or unmet spiritual needs have a negative effect on patients’ health outcome [10]. When patients with advanced cancer find it difficult to overcome these symptom burdens, more work is needed to understand the possible effects of unmet spiritual needs [11].

Spirituality is an essential element of person-centered care and a critical factor in the way patients with cancer cope with their illness, from diagnosis through treatment, survival, recurrence, and dying [12]. Stress, illness, and impending death can trigger deep existential issues. Spirituality is helpful for patients coping with worsening physical symptoms [13], and, psychological distress [14], and to be helps patients find value and meaning in life. Spiritual needs are the inner needs of humans, which reflect spirituality at different levels [15], and the aspirations an individual required or wanted to find meaning and purpose in life [11]. However, the definition of spiritual needs is complex. Several authors assumed spiritual needs reflects the complexity of human experience [15], that cannot be separated from the physical, emotional, social, or cognitive aspects of a person [16].

Although mounting empirical evidence describes the spiritual needs of patients with cancer, studies on such needs are not comprehensive [17]. First, consensus has not been reached on the definition of spiritual needs, and the most commonly used definition is as follows: the expectations and needs of each person to find meaning, value and purpose in life, and to experience the relationship between oneself and the present, others, beliefs, and nature [18]. Second, data from previous meta-analyses suggest that spiritual needs may influence physical, mental, and social health outcomes among cancer survivors [19, 20]. The most relevant studies, in patients with cancer, have focused on the relationship between spiritual well-being, quality of life (QOL), and hope. However, these findings have not been well characterized or tailored to patients’ spiritual needs based on clinical or treatment-specific factors [21–23]. As a response, we expect to further define the connections between the influencing factors and assess the content covered by spiritual needs. Therefore, the correct identification of the types of spiritual needs and their influencing factors in cancer patients, and comprehensively improving the medical staff's awareness of the patients' spiritual needs are still an urgent problem to be solved.

As medicine has moved toward a more inclusive biopsychosocial-spiritual model, Hiatt (1986) recommended that spirituality should be integrated into healthcare practice models. He proposed that the bio-psycho-social model needed to include the spiritual dimension, because it is concerned with meaning in life, it is also a determinant of health-related attitudes [24]. Spirituality is also one of the fundamental dimensions of quality of life; genuine holistic health care must address the totality of the patient, that is, his or her relationship with the physical, psychological, social, and spiritual aspects [25]. Meeting patients' spiritual needs increase their feelings of hope, comfort, and meaning across the cancer care continuum [26]. Based on the biopsychosocial spiritual model, spirituality can help patients advance their physical health, have positive attitudes, communicate and share with others, and have religious beliefs and hope [27]. Therefore, we made modifications according to the bio-psycho-social mental care model [12] and the conceptual model of palliative care needs [28]. In this study, based on the aforementioned previous models, we developed a biopsychosocial-spiritual model, by investigating the current state of the spiritual needs of patients with advanced cancer and exploring the relationship among the factors affecting their spiritual needs. The development of this model and its ability to provide a deeper understanding of these needs and their influencing factors is only a first step; our future outlook is for this model to be used in the integration of targeted spiritual care support for patients with cancer.

## Methods

### Study design and setting

**Fig. 1:**
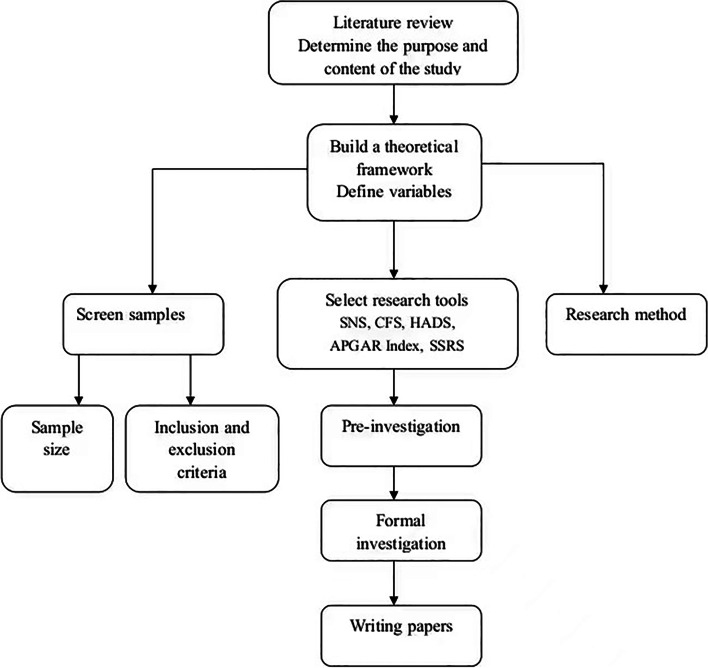
Flow diagram of the progress through the phases of study design and setting

This hospital-based cross-sectional quantitative study was conducted on 200 oncology inpatients, between December 2020 and June 2022, in the oncology departments of four tertiary first-class hospitals in Shandong Province. After obtaining informed consent from the patients and their families, the investigators distributed the questionnaires, and the respondents either filled out the questionnaires themselves or with the assistance of the researchers. After completion, the questionnaires were immediately collected. The researchers checked the questionnaires and asked the patients to correct and supplement it if the filling was incorrect or incomplete (Fig. 1). A total of 210 questionnaires were distributed in this study, of which 200 were returned. The effective response rate of the questionnaire was 95.2%.

**Fig. 2:**
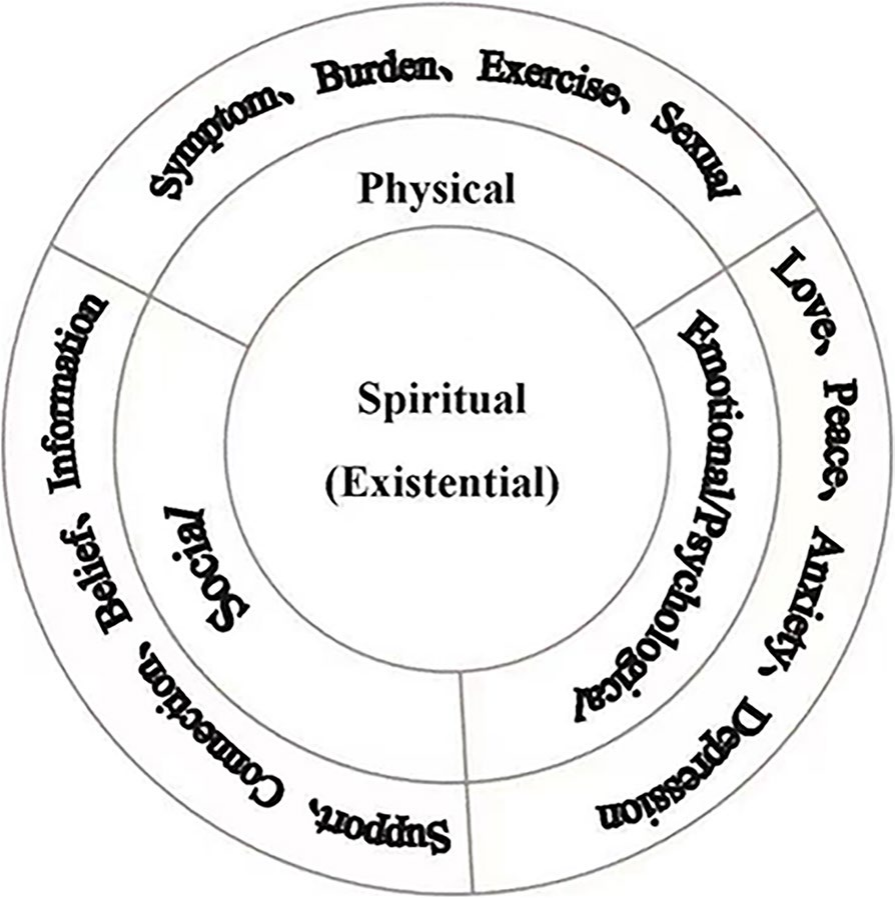
The modified biopsychosocial-spiritual model of care: whole-person care

We analyzed the factors influencing the spiritual needs of patients with advanced cancer according to the Modified Biopsychosocial-Spiritual Model (Fig. 2). This new model shows that spirituality is the core, and the outer layer, that is, the physical, psychological, and social levels, constitutes a whole person. In the process of promoting health through self-regulation, human beings must adjust their body (physiology and living ability), function (physical symptoms, such as fatigue and pain), mood (happiness, anger, love, and sadness), and behavior (cognition). These four levels are interrelated to form a whole that promotes overall health.

### Participants

The study population consisted of 200 oncology inpatients diagnosed with stage III–IV cancer.

To minimize selection bias, eligible patients were identified from the list provided by the oncology department. The list was forwarded to the assigned participants, who were investigated and surveyed after verbal explanations of the study’s aim, benefits, and importance, and obtaining informed consent from the patients. Patients were also provided with detailed information along with a questionnaire pack. The researcher asked the patients to take their time to decide whether they wanted to participate in this study. The researcher informed the patients of the voluntary nature of their participation in the study, their right to withdraw at any time, and that non-participation would not affect the care they received. The anonymity of their responses was guaranteed.

### Inclusion and exclusion criteria

The inclusion criteria were as follows: 1) cancer diagnosis by pathology/cytology, 2) diagnosed with TNM stage III–IV, 3) age > 18 years, 4) awareness of their diseases, 5) normal communication ability, and 6) voluntary participation. Patients with severe mental illness or disturbance of consciousness, and those with severe cognitive impairment were excluded. This study was approved by the Ethics Committee of the Fourth People's Hospital of Jinan City, and all participants signed an informed consent form and voluntarily participated in this study.

### Sample size

The required sample size was calculated using PASS software (version 15.0). Because multiple regression analysis was used to explore the influencing factors, the effect value method of multiple regression in PASS was used to estimate the minimum sample size for multiple regression analysis, which resulted in a minimal sample size of 72 participants for an effect value of 0.35, a power of 90%, and a statistical significance level of 5%.

Based on previous research results [29], in a Chinese study, considering that the average non-response rate was reported to be 9.0% of 200 patients, this study sample was increased to 210 patients to compensate for non-respondents, and 200 patients were included in the final analysis.

## Measures

### Questionnaire for General Information

Through a literature review, we designed a questionnaire for general information according to the research content and goals, including sex, age, religious belief, marital status, work status, number of hospitalizations, cancer types, gastrointestinal symptoms, pain, sleep quality, and exercise intensity.

### Chinese version of the spiritual needs scale

The Spiritual Needs Scale (SNS) developed by the Korean scholar Yong [30] is a spiritual needs evaluation tool in the context of Eastern culture. In 2018, Chinese scholar Cheng Qinqin [29] translated the scale into Chinese, and its reliability and validity were tested among Chinese patients with cancer. The Chinese version of SNS can be used to assess the spiritual needs of domestic patients with cancer. The scale has five dimensions: love and connection, hope and peace, meaning and purpose, relationship with the supernatural, and acceptance of death, with a total of 23 items. An example item is “Gain inner peace by overcoming the pain caused by treatment”. The total score ranges from 23 to 115, and patients with cancer are asked to describe their specific needs and choose a response on a 5-point scale where 1 = “not at all,” 2 = “seldom,” 3 = “sometimes,” 4 = “often,” and 5 = “a great deal.” A higher score indicates a greater need. In this study, Cronbach’s alpha was calculated as 0.86 for the whole scale, and for the five dimensions, love and relationship was 0.60, hope and peace was 0.59, meaning and purpose was 0.79, connection with the supernatural was 0.82, and death acceptance was 0.66, indicating a good reliability.

### Cancer fatigue scale

The Cancer Fatigue Scale (CFS) was designed by Okuyama [31] and has been used to assess the fatigue status of patients with cancer in the recent past. The scale includes three dimensions—physical, emotional, and cognitive fatigue—with a total of 15 items. An example item is “Does your body feel tired?”. The total score ranges from 0 to 60, with a higher score indicating a more severe level of fatigue. We used a CFS of > 18 points as the cutoff value for fatigue. Chinese scholars Zhang Fengling et al. [32]. translated the CFS into Chinese. In this study, Cronbach’s alpha was 0.70 for the entire scale and 0.87, 0.79, and 0.72 for the three dimensions, respectively, indicating good reliability.

### The hospital anxiety and depression scale

The Hospital Anxiety and Depression Scale** (**HADS) was developed by Zigmond and Snaith in 1983 [17] and has been widely used to assess depression and anxiety symptoms in hospitalized patients. The Chinese version of the HADS was translated and revised by Ye Weifei et al., and includes two subscales of anxiety and depression, with a total of 14 items. Higher scores on the scale indicates that patients’ symptoms of anxiety or depression are more severe. An example item for the hospital anxiety Scale is “I'm a bit restless, as if I feel compelled to move”. “I'm still interested in the things that I used to be interested in” is an example of an item in the hospital depression scale. In this study, Cronbach's α coefficients of the anxiety and depression scales were 0.82 and 0.76, respectively, indicating good reliability.

### Family APGAR index

Based on family functional characteristics, this questionnaire was designed in 1978 by Dr. Smilkstein of the University of Washington in Seattle (UW) [33]. This scale has been widely used for its simplicity and easy implementation, An example item is “When I encounter difficulties, I can get satisfactory help from my family”. The scale was introduced in China by Professor Lv Fan in 1995 and has gradually been applied domestically. In this study, Cronbach's α coefficient of the scale was 0.87, indicating good reliability.

### Social support revalued scale

The Social Support Revalued Scale (SSRS) was compiled by Professor Xiao Shuiyuan [34] in 1986. It contains 10 items in total, which are divided into three dimensions: objective support, subjective support, and social support utilization. An example item is “How many close friends do you have who can give you support and help”. The score on the scale was divided into three levels: low, medium, and high. A total score of low (≤ 22 points), medium (23–44 points), and high (45–66 points) indicates less, moderate, and satisfactory social support, respectively. A higher score indicated better social support. In this study, Cronbach’s alpha was calculated as 0.71 for the entire scale and between 0.44 and 0.57 for the three dimensions.

### Statistical analysis

SPSS 24.0 was used for statistical analyses of the data. Descriptive statistical analysis was performed on patients' general information, spiritual needs, cancer fatigue, anxiety and depression, clinical physiology and psychological symptoms, family care index, and social support. The influence of general patient information on spiritual needs was analyzed by the t-test or variance analysis, and Pearson correlation analysis was used to explore the correlation between cancer-related fatigue, anxiety and depression, family care index, social support, spiritual needs, and spiritual needs as the dependent variable to include influencing factors for multiple linear regression analysis. *p* < 0.05 was considered to be statistically significant.

## Results

### General information about patients with advanced cancer

A total of 200 patients were included in this study, of whom 121 were female (60.50%) and 79 were male (39.50%). Most patients were 41–65 years old (63.00%), with an average age of (58.10 ± 11.63) years. Digestive tract tumors were the most common cancer type (66 cases, 33.00%), followed by lung cancer (52 cases [26.00%]), breast cancer (43 cases [21.50%]), and gynecological tumors (17 cases [8.50%]).

To examine the relationships between the general characteristics of the participants and their spiritual needs, t tests (for associations with gender, religiosity, and exercise intensity) and ANOVA (for age, marital status, work status, frequency of hospitalizations, cancer type, gastrointestinal symptoms, pain level, and sleep quality) were conducted. Among the general information, the results indicated that spiritual needs of participants differed significantly based on age (*p* = 0.019), marital status (*p* = 0.002), religious beliefs (*p* = 0.001), and exercise intensity (*p* = 0.010) were statistically significant (*p* < 0.05) (Table 1).

**Table 1:** Univariate analysis of general information and spiritual needs of patients with advanced cancer (*N* = 200)

**Characteristics**	**Number of cases (percentage)**	**Scores of spiritual needs(points, ‾x ± s)**	**t/F**	***p* value**
**Gender**				
Male	79(39.50)	82.27 ± 14.11^1)^	-1.301^1)^	0.195
Female	121 (60.50)	85.24 ± 16.82^1)^		
**Age**				
≤ 40 years old	14 (7.00)	87.93 ± 16.98	3.386^2)^	0.019
41–50 years old	44 (20.00)	90.00 ± 13.78		
51–60 years old	45 (22.50)	81.91 ± 17.36		
≥ 61 years old	97 (48.50)	81.81 ± 15.23		
**Marital status**				
Married	6 (3.00)	96.50 ± 18.56	6.427^2)^	0.002
Unmarried	175 (87.50)	82.62 ± 15.21		
Divorced or Widowed	19 (9.50)	93.47 ± 16.61		
**Work status**				
On duty	21 (10.50)	88.43 ± 13.72	0.901^2)^	0.408
Retired	81 (40.50)	83.36 ± 14.90		
Unemployed	98 (49.00)	83.71 ± 16.97		
**Religious belief**				
No	171 (85.50)	82.42 ± 15.09^1)^	3.422^1)^	0.001
Yes	29 (14.50)	93.75 ± 16.94^1)^		
**Hospitalization**				
1–2 times	35 (17.50)	83.89 ± 15.80	0.032^2)^	0.968
3–4 times	35 (17.50)	84.69 ± 17.32		
More than 5 times	130 (65.00)	83.95 ± 15.55		
**Cancer type**				
Head and neck tumor	9 (4.50)	83.78 ± 17.33	0.640^2)^	0.670
Lung cancer	52 (26.00)	83.81 ± 15.02		
GI tract cancer	66 (33.00)	83.61 ± 14.47		
Breast cancer	43 (21.50)	87.40 ± 15.82		
Gynecologic Oncology	17 (8.50)	81.00 ± 22.58		
Others	13 (6.50)	80.62 ± 15.55		
**Gastrointestinal symptoms**				
Poor appetite	99 (49.50)	86.96 ± 15.14	2.534^2)^	0.058
Nausea	70 (35.00)	81.36 ± 16.68		
Stomatitis	10 (5.00)	77.10 ± 14.07		
Constipation	21 (10.50)	82.76 ± 15.18		
**Pain level**				
Painless	97 (48.50)	81.45 ± 13.58	1.848^2)^	0.140
Mild pain	74 (37.00)	85.97 ± 16.20		
Moderate pain	23 (11.50)	87.65 ± 20.08		
Severe pain	6 (3.00)	89.00 ± 23.71		
**Sleep quality**				
Difficulty falling asleep	102 (51.00)	82.58 ± 16.29	0.705^2)^	0.590
Insomnia-late	30 (15.00)	86.07 ± 13.09		
Restless sleep	25 (12.50)	87.24 ± 14.31		
Medication needs for sleep	28 (14.00)	83.18 ± 18.16		
Insomnia	15 (7.50)	86.53 ± 16.04		
**Exercise intensity**				
Moderate-intensity	79 (39.50)	87.61 ± 14.47^1)^	2.593^1)^	0.010
Mild intensity	121 (60.50)	81.75 ± 16.31^1)^		

^1^t test

^2^ANOVA

### Current spiritual needs of patients with advanced cancer

The average spiritual needs score of patients with advanced cancer was 84.07 ± 15.84 points (ranges from 38.00 to 115.00), which was a relatively high total score. The score of love and relationship was 20.37 ± 3.76 points (ranges from 8.00 to 25.00), score of hope and peace was 15.94 ± 3.47 points (ranges from 5.00 to 20.00), score of meaning and purpose was 23.21 ± 5.58 points (ranges from 6.00 to 30.00), score of connection with the supernatural was 8.00 ± 4.14 points (ranges from 3.00 to 15.00), and score of death acceptance was 16.55 ± 4.86 points (ranges from 5.00 to 25.00). To average the score difference caused by different entries in each dimension, divide the score of each dimension by the corresponding number of entries, and the result shows that among the five dimensions, love and relationship score 4.07 ± 0.75 (range 8.00–25.00) has the highest score, and the connection with supernatural score 2.67 ± 1.38 (range 3.00–15.00) has the lowest score. The scores for each dimension are presented in Table 2.

**Table 2:** The score of spiritual needs of patients with advanced cancer

	**Number of items**	**Minimum value**	**Maximum value**	**Mean value**	**Standard deviation**
**Spiritual needs**	23	38.00	115.00	84.07	15.84
Love and relationship	5	8.00	25.00	20.37	3.76
Hope and peace	4	5.00	20.00	15.94	3.47
Meaning and purpose	6	6.00	30.00	23.21	5.58
Connection with the supernatural	3	3.00	15.00	8.00	4.14
Death acceptance	5	5.00	25.00	16.55	4.86

### Correlation analysis between spiritual needs and cancer-related fatigue, anxiety and depression, family care index, and social support in patients with advanced cancer

Bi-variable analyses showed that the spiritual needs of patients were significantly correlated with cancer-related fatigue, depression, and social support. Spiritual needs were significantly positively correlated with cancer-related fatigue (*r* = 0.191, *p* < 0.007), significantly positively correlated with social support (*r* = 0.203, *p* < 0.004), and significantly negatively correlated with depression (*r* = -0.173, *p* < 0.014) (Table 3).

**Table 3:** Correlation analysis between spiritual needs and influencing factors of patients with advanced cancer

Influencing factors	*r* value	*p* value
Cancer‐related fatigue	0.191	0.007
Anxiety	-0.071	0.315
Depression	-0.173	0.014
Family care index	0.037	0.607
Social support	0.203	0.004

^§^*p*-value has been calculated using Pearson correlation analysis

### Multivariate analysis of spiritual needs of patients with advanced cancer

Multiple linear regression was performed with spiritual needs as the dependent variable and age, marital status (divorced or widowed), exercise intensity, religious beliefs, cancer-related fatigue, depression, and social support as independent variables. The results showed that marital status (divorced or widowed), religious beliefs, cancer-related fatigue, and social support significantly affected spiritual needs. B unstandardized estimated change of marital status (divorced or widowed) was 8.531 [95% confidence interval {CI} (1.512–15.550)], B unstandardized estimated change of religious beliefs was 8.675 [95% CI (2.876–14.473)], B unstandardized estimated change of cancer-related fatigue was 0.498 [95% CI (0.251–0.744)], and B unstandardized estimated change of social support was 0.364 [95% CI (0.101–0.628)]. Further details are provided in Table 4. The significance of the equation was tested using analysis of variance. The results showed that F = 8.970 and *p* < 0.001, indicating the multiple linear regression equation was statistically significant. The fitted regression equation was evaluated using the complex phase relationship number R and the determination coefficient R^2^, *R* = 0.503, *R*^*2*^ = 0.253, and adjusted *R*^*2*^ = 0.214. Results indicated that religious beliefs, cancer-related fatigue, and social support accounted for 21.4% of the total variation in spiritual needs.

**Table 4:** Multiple linear regression of influencing factors of spiritual needs of patients with advanced cancer

	**Unstandardized coefficients**	**95% CI**	**t**	***p* value**	**VIF**
(constant)	49.514	31.528—67.499	5.396	0.000	-
Age(41–50 years)	1.855	-6.980—10.689	0.411	0.681	3.536
Age(51–60 years)	-3.649	-12.488—5.189	-0.809	0.419	3.597
Age(≥ 61 years)	-6.430	-14.951—2.090	-1.479	0.141	4.787
Marital status (Unmarried)	4.251	-8.093—16.596	0.675	0.501	1.171
Marital status (Divorced or Widowed)	8.531	1.512—15.550	2.382	0.018	1.118
Religious belief	8.675	2.876—14.473	2.932	0.004	1.101
Exercise intensity	3.322	-0.885—7.529	1.547	0.123	1.117
Cancer-related fatigue	0.498	0.251—0.744	3.955	0.0001	1.162
Depression	-0.259	-0.775—0.257	-0.985	0.326	1.383
Social support	0.364	0.101—0.628	2.710	0.007	1.344

*R* = 0.503, *R*^*2*^ = 0.253, adjust *R*^*2*^ = 0.214, F = 6.408

**Fig. 3:**
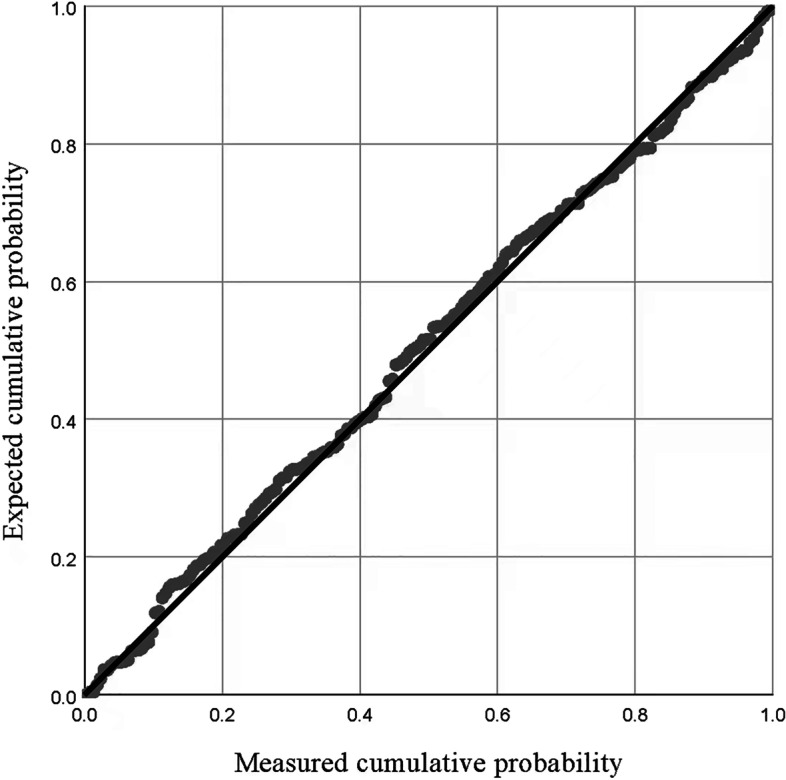
Normal P-P Plot of Regression Standard Residual of Dependent variable: Spiritual Need

**Fig. 4:**
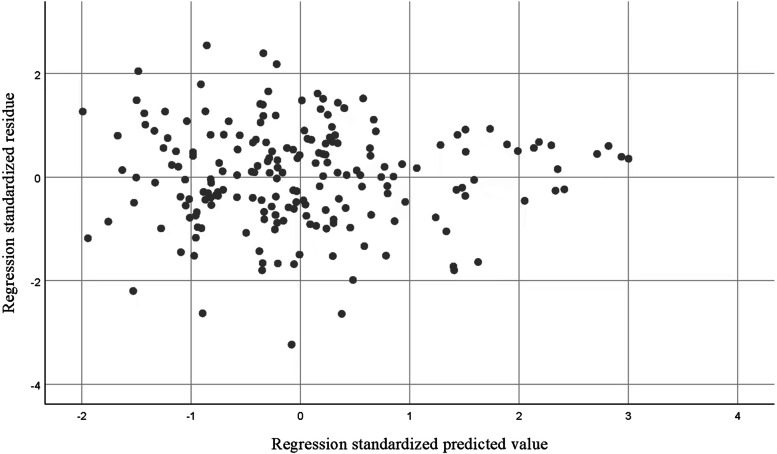
Scatter plot of Regression Standard Residual of Dependent variable: Spiritual Need

Figure 3 show the normal distribution of residuals. The regression model analyzed for linearity (linear relationship between independent and dependent variables), homoscedasticity (variance of the residuals consistent at each level of the independent variable), and multicollinearity (variance inflation factor below 5) (Fig. 4).

## Discussion

One of our study aims was to explore the status of patients’ spiritual needs in northern China. The overall spiritual needs scores of patients with advanced cancer in this survey were relatively high, with an average score of 84.07 ± 15.84, and were closely related to prolonged disease course, repeated hospitalisation, and worsening patient physical condition. Our results showed that love and connection had the highest scores related to the spiritual needs of patients with advanced cancer, which inconsistent with previous research of Moadel et al. [8]. Spiritual need has been defined as “the needs and expectations that humans have to find meaning, purpose, and value in their life” [35]. However, our results showed that patients identified love and connection as an important spiritual need, which indicated that they had a stronger need to express love and receive care from others. Patients with advanced cancer require more emotional and family support compared to other patients.

Regarding specific sociodemographic data, the results showed significant differences in the spiritual needs and general characteristics of patients with advanced cancer, including age, marital status, religious beliefs, and exercise intensity. The results of this study showed that patients’ spiritual needs were significantly correlated with their marital status (*p* < 0.05), family relationships was a major spiritual need identified by patients in line with the findings of previous studies of Murray et al. [8]. Marital status is a core factor related to patients gaining a sense of meaning and happiness. Chinese people have a strong traditional concept of marriage, which plays an important role in life and social development [36]. It is noteworthy that the findings of this study also revealed significant differences in spiritual needs and exercise intensity (*p* < 0.05), which is consistent with the results of Mojgan et al. [37]. Some studies have reported moderate-intensity exercise can improve physical and emotional functioning in patients by increasing the level of self-efficacy, which ultimately leads to an overall improvement in health status [38]. Nurses can determine the best exercise regimen tailored to each patient’s condition, improve spiritual well-being, and meet spiritual needs. However, in the present study, patients’ spiritual needs were not significantly correlated with cancer-related symptoms such as pain, insomnia, and nausea.

Religion and spirituality are both important resources for responding to cancer [39]. Previous studies have shown that religious beliefs can affect the spiritual needs of patients, and patients with religious beliefs have a more peaceful attitude toward death and are more willing to express their needs for death [40], showing that religious beliefs are the main influencing factors of spiritual needs, which is consistent with the results of Shi Yan et al. [41]. Yang Qing et al. [42]. found in their qualitative research that there were two types of spiritual needs in patients with advanced cancer: the need to respect religious beliefs and the need to remember and reflect on oneself. Patients with religious beliefs tend to seek transcendence for support, hope, optimism, and inner strength [43]. Conversely, for patients with non-religious backgrounds, seeking the meaning/peace dimension of spirituality had the strongest positive effects on the overall QOL as well as physical and mental health [5]. Among patients with advanced cancer in our study, 172 (86.00%) had no religious beliefs. Some patients said that they would like to chat or write to express their insights about life or life experiences for comfort. Consequently, medical staff should searching for meaning in life through religious or cultural explanations to help them expressing beliefs more freely; respect them with empathy; use language that resonates with them; and encourage patients to find their inner peace, thereby improving their quality of life.

Cancer-related fatigue (CRF), the most common physical complication of cancer, is defined as “a distressing and persistent, subjective sense of physical, emotional and/or cognitive tiredness related to cancer or cancer treatment.” [44]. In the present study, the cancer-related fatigue score of patients with advanced cancer was 45.43 ± 8.41, indicating severe fatigue. Our results confirmed a significant correlation between spiritual needs and cancer-related fatigue (*p* < 0.05), this finding consistent with previous reports of Lewis et al. [45]. The research shows that when patients with cancer are in distress and experience physical or mental exhaustion, they start thinking about the meaning of life, the value of existence, interpersonal relationships, the need for hope, and other spiritual needs [46]. In their study of 200 patients with cancer, Lewis et al. [45] reported that mental fatigue was negatively correlated with spiritual health. Liu X et al. [47]. observed that reducing patients’ negative emotions improved their emotional tiredness. In many cases, physical symptoms and the accompanying emotional cognition are inseparable. However, in clinical practice, when patients complain about “physical and mental exhaustion”, we focus mainly on fatigue itself and related symptoms while ignoring the patients’ mental exhaustion, which in turn negatively affects their spiritual health. Therefore, nursing staff can alleviate symptoms of physical fatigue by maintaining proper exercise, improving sleep quality, and providing patients with adequate nutritional support. Moreover, providing the necessary emotional support to patients and, reducing their negative emotions can relieve their emotional fatigue. Simultaneously, we can improve cognitive fatigue and provide patients with an effective way to relieve fatigue, thereby enhancing patient compliance and providing support to obtain an ideal quality of life.

In several cases, spiritual needs correlate with psychological health-related indicators, such as anxiety, depression [48]. In this study, the depression score of patients with advanced cancer was 7.12 ± 4.47 (ranges, 0.00–18.00), and the incidence of depression was 53.5%. The results of this study showed that patients’ spiritual needs were significantly negatively correlated with depression (*p* < 0.05), this finding consistent with studies of Douglas and Daly [25]. Depression predicts the loss of motivation for the meaning of life in patients, who often feel sad and hopeless, are taciturn, lose interest in everything, and even lose their life meaning. Studies have shown that good spiritual health can reduce the impact of depression on patients with cancer and improve their mental health [49]. Therefore, when focusing on patients’ spiritual needs, medical staff should consider the potential impact of negative emotions on spiritual needs and actively carry out psychological counseling interventions [42]. For example, to relieve depressive symptoms and improve the spiritual well-being of patients, medical staff should intently listen to the patients voice with empathy, orient them toward the meaning and value of life, have meaning-oriented conversations, and for stress reduction, incorporate meditation and mindfulness into the patients’ therapy regimen.

Human connections are perceived as a prerequisite for providing effective spiritual care [50]. This study showed that the patients obtained a social support score of 36.20 ± 8.52 (ranges from 13.00 to 62.00), indicating satisfactory social support. Among the dimensions of the social support scale, subjective support has the highest score of 20.37 ± 5.24 (ranges from 8.00 to 32.00); followed by objective support score of 9.39 ± 3.66 (ranges from 1.00 to 23.00); and the utilization score is the lowest with 6.45 ± 2.65 (ranges from 3.00–19.00). The result this study show that social support is one of these predictors of spiritual needs, which supported the findings of previous studies of patients with cancer [37]. Studies have shown that quality interpersonal relationships and social support are given it gave the patient strength and spiritually support [28], which are important for Chinese people especially. In this study, we found that patient’s needs were met, which is related to the objective support they received and more subjective support they sought. For patients with cancer, social support is more important for psychological counseling and spiritual encouragement, followed by life care and economic support. Good social support especially the support and encouragement of spouses or children, have obvious positive effects on the recovery of cancer patients, which can greatly affect their confidence in treatment and their hope of life [51]. Medical staff can help patients with cancer face their disease positively and help them find hope and meaning in life again by improving objective support, that is, including listening, encouraging, organizing patient clubs, reading clubs, and other forms of social support.

### Limitations

This cross-sectional study included only inpatients from the oncology departments of four hospitals in Shandong Province. The sample size was small. Outpatients were not included in the study; therefore, future studies should increase the sample size. The sample may be unrepresentative, since respondents experienced different and diverse range of symptoms and differed culturally. Given these limitations, future studies of multidisciplinary clinical pathways are highly recommended.

Furthermore, there is still insufficient research on the factors that influence the flexible and changeable needs of patients with cancer in domestic situations. The development of practical and localized measurement tools that cater to the changeable needs of patients with cancer is encouraged, as they can provide evidence-based clinical guidance for comprehensively and accurately evaluating the flexible and changeable needs of cancer patients.

## Conclusion

In this study, patients with advanced cancer had higher levels of spiritual need. Based on the Biopsychosocial-Spiritual Model, the results showed that cancer-related fatigue, social support, and religious beliefs were factors influencing the spiritual needs of patients with advanced cancer. The analysis of influencing factors can provide a "handle" for clinical nurses to identify the inherent characteristics and correlations of patients' spiritual needs. Currently, however, there is a lack of domestic assessment tools for spiritual needs in China. People are often reluctant to mention hospice care or spiritual needs, which may make the patient feel bad about giving advice in a direct or explicit way. Nurses or care providers should identify and perceive patients’ spiritual needs through their utterances [52]. However, the author found that regardless of whether the relevant nursing measures were implemented or not, after completing this questionnaire survey, patients fed back their feelings, and most patients felt relaxed, comfortable, and experienced emotional release in this study. Therefore, we can confidently ascertain that the SNS can be applied to patients with cancer in the Chinese context. Further verification is needed regarding whether we can meet the spiritual needs of patients by means of communication to help them release their emotions, how to further symptom management to alleviate their spiritual pain, and how to change their cognition and improve their subjective initiative in the spiritual care.

## Supplementary Information

**Additional file 1. **Raw Data-Spiritual Needs of Patients with Advanced Cancer.

## Data Availability

All data generated or analyzed during this study are included in this published article and its supplementary information file.

## Abbreviations

SNS: Spiritual Needs Scale
CFS: The Cancer Fatigue Scale
HADS: The Hospital Anxiety and Depress Scale
SSRS: Social Support Revalued Scale
CRF: Cancer-related fatigue
ANOVA: Analysis of variance
QOL: Quality of life
